# Ecological Genetics of Chinese Rhesus Macaque in Response to Mountain Building: All Things Are Not Equal

**DOI:** 10.1371/journal.pone.0055315

**Published:** 2013-02-06

**Authors:** Shan-Jin Wu, Jing Luo, Qing-Qing Li, Yan-Qin Wang, Robert W. Murphy, Christopher Blair, Shi-Fang Wu, Bi-Song Yue, Ya-Ping Zhang

**Affiliations:** 1 Laboratory for Conservation and Utilization of Bio-resources, School of Life Science, Yunnan University, Kunming, China; 2 Sichuan Key Laboratory of Conservation Biology on Endangered Wildlife, College of Life Sciences, Sichuan University, Chengdu, Sichuan, China; 3 State Key Laboratory of Genetic Resources and Evolution, Kunming Institute of Zoology, the Chinese Academy of Sciences, Kunming, China; 4 School of Life Sciences, Yunnan Normal University, Kunming, China; 5 Centre for Biodiversity and Conservation Biology, Department of Natural History, Royal Ontario Museum, Toronto, Ontario, Canada; 6 Department of Biology, Duke University, Durham, North Carolina, United States of America; Lund University, Sweden

## Abstract

**Background:**

Pliocene uplifting of the Qinghai-Tibetan Plateau (QTP) and Quaternary glaciation may have impacted the Asian biota more than any other events. Little is documented with respect to how the geological and climatological events influenced speciation as well as spatial and genetic structuring, especially in vertebrate endotherms. *Macaca mulatta* is the most widely distributed non-human primate. It may be the most suitable model to test hypotheses regarding the genetic consequences of orogenesis on an endotherm.

**Methodology and Principal Findings:**

Using a large dataset of maternally inherited mitochondrial DNA gene sequences and nuclear microsatellite DNA data, we discovered two maternal super-haplogroups exist, one in western China and the other in eastern China. *M. mulatta* formed around 2.31 Ma (1.51–3.15, 95%), and divergence of the two major matrilines was estimated at 1.15 Ma (0.78–1.55, 95%). The western super-haplogroup exhibits significant geographic structure. In contrast, the eastern super-haplogroup has far greater haplotypic variability with little structure based on analyses of six variable microsatellite loci using Structure and Geneland. Analysis using Migrate detected greater gene flow from WEST to EAST than vice versa. We did not detect signals of bottlenecking in most populations.

**Conclusions:**

Analyses of the nuclear and mitochondrial datasets obtained large differences in genetic patterns for *M. mulatta*. The difference likely reflects inheritance mechanisms of the maternally inherited mtDNA genome versus nuclear biparentally inherited STRs and male-mediated gene flow. Dramatic environmental changes may be responsible for shaping the matrilineal history of macaques. The timing of events, the formation of *M. mulatta*, and the divergence of the super-haplogroups, corresponds to both the uplifting of the QTP and Quaternary climatic oscillations. Orogenesis likely drove divergence of western populations in China, and Pleistocene glaciations are likely responsible for genetic structuring in the eastern super-haplogroup via geographic isolation and secondary contact.

## Introduction

The Pliocene uplifting of the Qinghai-Tibetan Plateau (QTP) and Quaternary glaciation may be the primary drivers of genetic divergence and patterning of the Asian biota. With a mean elevation of about 4000 m above sea level and an area of approximately 2.5 million km^2^, the QTP is the largest high-elevation plateau on Earth. Three phases–A, B, and C, and occurring about 3.6 million years ago (Ma), 2.6 Ma, and 1.7 Ma, respectively–characterize the last orogenic push of the QTP [1]. This orogenesis is associated with the formation of Asian monsoons, the beginning of Chinese loess (aeolian sedimentary deposits), and the appearance of the Yellow (Huanghe) River [1]. Drastic environmental changes in Southeast Asia and especially in China are a response to these events. Dramatic climatic oscillations of the Quaternary Period profoundly affected the current distribution of most living organisms and their genetic structuring [2], [3]. At times of Pleistocene glacial maxima, ice sheets did not cover most of East Asia [3], [4], yet glaciation and its associated climatic oscillations profoundly affected some biota [5], [6].

Geological and climatological events influenced speciation and spatial and genetic structuring, especially among ectotherms. However, much remains to be documented with respect to endothermic vertebrates. *Macaca mulatta*, the rhesus macaque, may be the most suitable model to investigate the relationship between the non-human primates and the genetic consequences of orogenesis. Its geographic range extends from Afghanistan in the west to the coast of the East China Sea in the east. The species occurs southwards from the Himalayas and central China to central India and central Indochina. This range, which exceeds that of all other primates except for humans [7], [8], is sufficiently extensive and old that major genetic structuring might have evolved [9].

Genus *Macaca* appears to have originated in North Africa approximately 5.5 Ma [10]. It seems to have dispersed to the Sawalik Hills of northern India, perhaps by a southern route, before 3 Ma and occupied Indonesia by 2 Ma [11]. Fossil evidence documents the presence of macaques in southeastern [12] and north-central [13] China before or in the early Pleistocene. Using the maternally, paternally, and biparentally inherited gene sequences, the evolutionary relationships of the *M. fascicularis* complex are well documented [14], [15], [16], [17], [18]. *Macaca fascicularis* forms the sister group of the other three species of *Macaca*, *M. cyclopis*, *M. mulatta*, and *M. fuscata* [19]. The *fascicularis* species group, which also contains rhesus and cynomolgus macaques, shared a common ancestor about 3.2 Ma [20], [21].

Diversity in the mitochondrial genome of rhesus macaques is reported from restriction fragment analyses [15], [20], DNA sequencing of mitochondrial genes [9], [22], [23], [24], [25], [26], [27], [28], [29], and microsatellites (short tandem repeats; STRs) [30], [31], [32]. Some studies using single nucleotide polymorphisms (SNPs) also exist [33], [34], [35], [36]. These studies provide evidence of regional variation within *M. mulatta*. For example, a large degree of genetic divergence occurs between Indian and Chinese populations. Smith and McDonough [9] characterize two major mtDNA haplogroups (CHIE and CHIW), which occur in eastern and western China, respectively. Satkoski et al. [28], [36] add a third haplogroup–ChiS–from southwestern China and Viet Nam.

Captive populations form the basis of most genetic assessments. Relatively few wild samples are assessed. Often these samples are missing specific locality data other than their provinces of origin [9], [29]. Satkoski et al. [28], [36] provided evidence that breeding stock was exchanged among the breeding centers in China, especially those in eastern China where their genetic density was lower. Thus, it is particularly important to use wild samples in the genealogical study of Chinese rhesus macaques. Further, male rhesus macaques are highly mobile and they often leave one troop to join others. In contrast, females rarely do so [37]. For *Macaca mulatta*, the maternal genetic structure, especially in the form of maternal genealogy, is likely to better reflect the history of troop dispersal and colonization than that of paternal history.

We assess the extent of genetic diversity and the evolutionary history of the most widely distributed nonhuman primate, the rhesus macaque, by using a large dataset of maternally inherited mitochondrial DNA gene sequences and nuclear DNA microsatellite data. We test a suite of hypotheses related to the geographic structure of genetic diversity in wild Chinese rhesus macaques based on mtDNA and microsatellite markers. Further, we test hypotheses on the relative roles played by dramatic environmental changes, in particular orogenesis of the Qinghai-Tibetan Plateau (QTP), and Quaternary climatic oscillations in shaping genetic structure.

## Materials and Methods

### Ethics Statement

The Anhui experimental rhesus monkey Center, Chengdu pingan animal Breeding and Research Center and Primate experimental animal center of KIZ have provide the provided the blood samples. Blood samples were salvaged from routine health checks for newly captured wild macaques at these breeding centers. We have obtained permission from the centers to analyze the blood samples for this study. These captures are usually as the founder populations of the breeding populations. And These captures of rhesus monkey were authorized by the specific government agency, for example, the Sichuan Department of Forestry, Yunnan Department of Forestry, or An Department of Forestry which is in charge of a certain sampling region. We collected the fecal samples from the Henan Taihang Macaque Nature Reserve, and the Zhangjiajie National Forest Park. We have obtained permission from the above administrative departments to observe the animal behavior of rhesus monkey and collect the stool samples. We also confirm that the behavioral observations did not impact the animals in any way.

### Sampling

**Figure 1:**
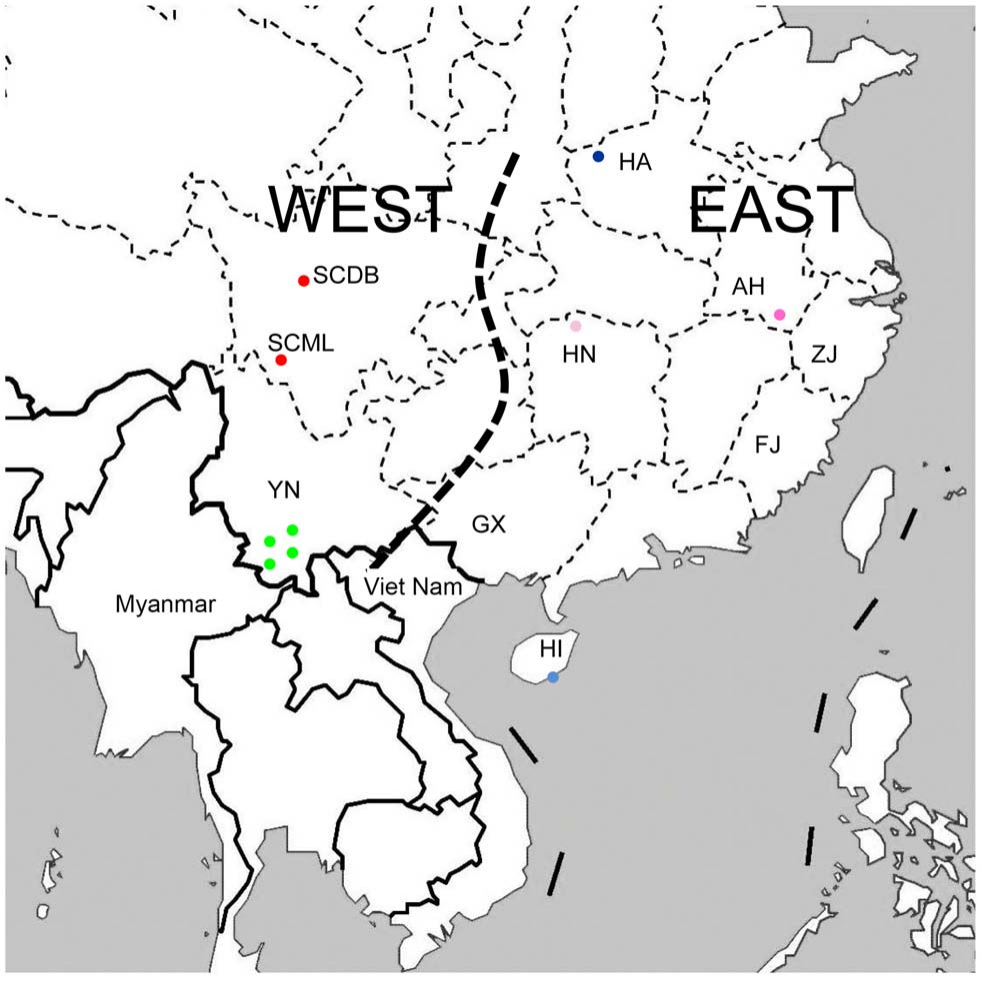
Map of sampling locations for the rhesus macaque, *Macaca mulatta*. Dots indicate specific locality data. SC = Sichuan (SCML = Muli; SCDB = Danba), YN = Yunnan (YNJG = Jinggu; YNSM = Simao ( = Pu’er); YNZY = Zhenyuang; YNMJ = Mojiang), HI = Hainan, HA = Henan, HN = Hunan, GZ = Guizhou, GX = Guangxi, FJ = Fujian, and ZJ = Zhejiang.

We collected 337 wild samples from most of the extant groups of *M. mulatta* across its entire range in China (Figure 1). Samples used for genetic analysis included feces (n = 78) and blood (n = 259). Blood samples were salvaged from routine health checks for newly captured wild macaques at breeding centers. Fecal samples, collected during direct behavioral observations, were stored in 95% ethanol. In order to avoid resampling of fecal material from the same individuals, we sampled each group once and on a specific day only. Each dropping was identified by size, shape, and color, and only one sample located <1.5 m apart was collected [38]. D-loop sequences from 168* M. mulatta* and 56 haplotypes from Sichuan used by Li et al. [29] were downloaded from GenBank. The downloaded data included 76, 21, 13, 109, and 5 sequences originating in China (Guangxi, 20; Sichuan, 56), Nepal, Myanmar, India, and Viet Nam, respectively. The detailed sample data were listed in Table S1.

### Laboratory Procedures

Blood samples were digested using proteinase K. This was followed by a standard 3-step phenol/chloroform extraction procedure. Fecal samples extracted using the 2CTAB/PCI protocol as modified by Vallet et al. [39]. DNA was then electrophoresed through an agarose gel to assess its quality and quantity. High-quality samples were diluted to ∼20 ng/µL in TE buffer (10 mM Tris-HCl, pH 8.0, 0.1 mM EDTA).

To reconstruct the matrilineal genealogy, three fragments from the mitochondrial genome were selected for sequencing. A partial fragment of the D-loop included 510 base pairs (bp) in length. Another two mitochondrial fragments included part of the cytochrome b (*Cytb*) gene (882 bp) and part of rRNA 16S (861 bp). Partial fragments of the hypervariable D-loop were initially sequenced for all 337 specimens. Subsequently, 16S and *Cytb* fragments were sequenced for a subset of samples (66 specimens) that represented the major haplogroups identified from the genealogical reconstruction of the D-loop data (Table S1). The data from the 16S and *Cytb* fragments were used to estimate divergences deeper than those revealed by the D-loop data. PCR cycling conditions for D-loop consisted of an initial denaturation of 5 min at 94°C, followed by 35 cycles (94°C denaturation for 1 min, 50°C annealing for 1 min, and for 1 min extension at 72°C). Final extension at 72°C was conducted for 10 min. We used the same procedure for *Cytb* and 16S but with annealing at 55°C and 59°C, respectively. Primers sequences were summarized in Table 1.

**Table 1:** Primers used in PCR and sequencing for the rhesus macaque, *Macaca mulatta*.

Locus	Primer name	Primer sequence	Size(bp)	Cited source
D-loop	1	5′-CCG CCC ACT CAG CCA ATT CCT GTT CT-3′	835	[9]
	2	5′-CCC GTG ATC CAT CGA GAT GTC TT-3′		[9]
	3	5′-TCC TAG GGC AAT CAG AAA GAA AG-3′	510	[90]
	4	5′-CCT GAA GTA GGA ACC AGA TG-3′		[90]
	5	5′-ATT CGT GCA TTA CTG CTA G-3′		this study
*Cytb*	6	5′-CAA CTA TAA AAG CAC CAA TGA C-5′	882	this study
	7	5′-GTT CGC TTC CAA TTC AGG T-3′		this study
	8	5′-CCT ACA CGA AAC AGG ATC AA-3′		this study
	9	5′-TGT AAG GTG AAG AAT CGT GTG-3′		this study
*16S*	10	5′-GTC CAA GGA GGA TTT AGC-3′	861	this study
	11	5′-TGT TAG GTA TCG GTG AGG-3′		this study
	12	5′-CGA AAC CAG ACG AGC TAC C-3′		this study
	13	5′-GGT AGC TCG TCT GGT TTC G-3′		this study

The amplified DNA fragments were purified via spin columns and sequenced using an ABI PRISM 3730 DNA analyzer following the manufacturer’s protocols. Sequences were determined in both directions for each individual. To preclude the inclusion of NUMTs, all fragments were submitted for BLAST searching [40] in GenBank to ensure that the required sequences had been amplified. Further, protein-coding nucleotide sequences were translated to amino acids using MEGA 4.1 [41] to check for premature stop codons.

We chose six previously reported tetranucleotide microsatellite loci (Table 2) to assess biparental gene flow. PCR procedures involved an initial denaturation at 94°C for 4 min followed by 30 cycles of 30 sec at 94°C, 40 sec at 52.1–60.0°C for annealing, 30 sec at 72°C, and a final extension at 72°C for 10 min. PCR products were diluted and mixed with LIZ 500 size standards. Microsatellites were obtained using an ABI PRISM 3730 DNA analyzer (ABI, USA). Allele scoring was performed using genemapper [42].

**Table 2:** Primer sequences used in the microsatellite study.

STR marker	chromosome	Primer sequence	Reference	Size range (bp)	N_A_	Annealing temperature
D7S1826	3	5′-[FAM]CATCCATCTATCTCTGTAATCTCTC-3′	[30]	93+115–155	12	58.4°C
		5′-TATTTAACACACCTGTCTCAATCC-3′				
D8S1106	8	5′-[FAM]TTGTTTACCCCTGCATCACT-3′	[30]	131–191	10	58.0°C
		5′-TTCTCAGAATTGCTCATAGTGC-3′				
D1S548	1	5′-[FAM]GAACTCATTGGCAAAAGGAA-3′	[91]	187–199	4	56.8°C
		5′-GCCTCTTTGTTGCAGTGATT-3′				
D5S1457	6	5′-[FAM]TAGGTTCTGGGCATGTCTGT-3′	[91]	103–135	9	58.4°C
		5′-TGCTTGGCACACTTCAGG-3′				
D11S2002	14	5′-[FAM]CATGGCCCTTCTTTTCATAG-3′	[30]	235–275	11	58.4°C
		5′-AATGAGGTCTTACTTTGTTGCC-3′				
D15S644	7	5′-[FAM]CCTTCATTGGCAGACTCACT-3′	[30]	174–262	23	58.4°C
		5′-GCAGACACCAAGATGATAACG-3′				

The fluorescent dyes, original references, and annealing temperatures used for rhesus macaque-derived markers, with the number of alleles (N_A_) and allele size range found in the 265 samples screened in this study are listed.^a,b.^

a The sequence of primers used to amplify each locus can be obtained from Research Genetics Inc.

b The chromosome number of the STR markers in *Macaca mulatta* was determined by e-PCR in the UniSTS database from NCBI.

### MtDNA Data Analyses

#### DNA sequence alignment

Alignments were first conducted using Clustal W [43] integrated into MEGA v.4.1 [41] while employing default parameters. When required, the initial alignments were subsequently adjusted by eye using MEGA v.4.1. Protein-coding nucleotide sequences were translated to amino acids using MEGA v.4.1 to confirm alignments. Gaps were considered to be a nucleotide variant (fifth state). Identical haplotypes were collapsed using DnaSP, v.5.0 [44].

#### Matrilineal relationships

For phylogenetic analyses, several species of macaques were selected as outgroup taxa. Two mitochondrial datasets were analyzed separately. First, the D-loop fragments alone, including 214 unique haplotypes plus the four outgroup taxa from *M. fascicularis*, *M. thibetana*, *M. sylvanus*, and *M. arctoides*, were subjected to phylogenetic analyses. The second dataset included all three mtDNA fragments for 67 haplotypes plus 12 outgroup taxa including *M. fascicularis*, *M. thibetana*, *M. sylvanus*, *M. arctoides*, *M. assamensis*, *M. maura*, *M. nigra*, *M. ochreata*, *M. pagensis*, *M. radiata*, and *M. silenus*.

A heuristic maximum parsimony approach was conducted for both datasets. All characters were unordered and equally weighted. The search was conducted using PAUP* v.4.0b10 [45] with 100 random stepwise additions and TBR branch swapping. Nonparametric bootstrap proportions (BSP) based on 1000 pseudoreplicates were used to infer nodal support.

For the first dataset, associations among the 214 D-loop haplotypes were also visualized by a full median-joining network [46], [47] with MP post-processing [48] as implemented in Network v.4.5 (www.fluxus-engineering.com/sharenet.htm). For the second dataset, phylogenetic relationships were also inferred using Bayesian inference as implemented in MrBayes v.3.1.2 [49]. The best-fitting nucleotide substitution model for each gene (16S:GTR+I+G, *Cytb*: GTR+G, D-loop: HKY+I+G) was selected based on the Akaike Information Criterion as implemented in Modeltest v.3.7 [50] using default parameters. Four Monte Carlo Markov chains (MCMC) were used and the dataset was run for 10 million generations and trees were sampled every 200 generations. The last 25% of the sampled trees were used to estimate the consensus tree and corresponding Bayesian posterior probabilities.

#### Isolation-by-distance and historical population analyses

Using the first dataset, we tested the null hypothesis of a correlation between genetic and geographical distances, i.e. isolation-by-distance (IBD), for the matrilines using both all ten sampling localities and the six western sites that had specific locality data (Figure 1). Approximate geographic coordinates were determined using Google Earth. Distances between localitied were estimated using the spherical distance between two points. The pairwise genetic differentiation values were assumed to measure the extent of DNA divergence between populations, and the significance was tested using 1,000 permutations with Arlequin v.3.11 [51]. Correlation of geographic and genetic distances was determined using Mantel’s permutation test with 10,000 permutations executed by IBDWS v.3.15 [52]. The strength of this relationship was determined by regressing all pairwise genetic differentiation values, *F_ST_*, against the corresponding log_10_ transformed geographical distance.

Historical population dynamics of the major haplogroups assigned in the median-joining network were examined with mismatch distribution analysis [53] as implemented in Arlequin v.3.11. This analysis compared the observed frequencies of pairwise differences of haplotypes with those expected under a single sudden expansion model [53]. An expected distribution under a model of sudden demographic expansion was generated with a total of 1000 permutations. Demographic stability has produced multimodal distributions, and unimodal patterns have occurred during sudden population expansion [54]. The raggedness index was expected to have a higher value in relatively stable populations. Tajima’s *D* [55] and Fu’s *Fs* statistics [56], calculated using Arlequin v.3.11, were also used to seek evidence of demographic expansions within individual haplogroups and subhaplogroups. Statistical significance was tested with 1000 permutations. The null hypothesis was that of a stable population. Additionally, we calculated the statistics of genetic diversity, neutrality tests, and mismatch distributions for the main populations of *Macaca mulatta* in China.

#### Estimation of divergence time

Divergence times were estimated using a Bayesian MCMC method implemented in beast v.1.5.3 [57], which employed a relaxed molecular clock approach [58]. A relaxed lognormal clock model of haplogroup variation and a Yule prior for branching rates was assumed. Divergence time estimations were based on the second dataset only. After removing indels in the outgroup taxa, the final alignment for divergence age estimations comprised 2,260 nucleotide positions. The dataset was partitioned and each optimal nucleotide substitution model was selected by using the Akaike Information Criterion as implemented in Modeltest v.3.7 [50].

As a calibration point, we used the divergence time of 5.5 Ma (95% confidence interval = 4.68–6.32 Ma) [10] for matrilines between *M. sylvanus* and the other Asian macaques. Instead of using hardbounded calibration points, we relied on published dates and specified a normal distribution prior, with a mean of 5.5 Ma and a standard deviation of 0.5 Ma. Two replicates were run for 100 million generations while sampling trees and parameters every 1,000 generations. The adequacy of using a 50% burn-in to generate MCMC trees and for convergence of all parameters was assessed visually using Tracer v.1.4.1 [59]. Subsequently, the sampling distributions of two independent replicates were combined using the software LogCombiner v.1.5.2 [54], and the resulting 100,002 samples were summarized and visualized using TreeAnnotator v.1.5.2 [54] and Fig Tree v.1.2 [60].

### STR Analyses

#### Statistical analyses

All loci were collected as an EXCEL file, and then converted into the computable files using convert [61]. We used Arlequin v.3.11 to check if the microsatellite data departed from Hardy-Weinberg expectations. Tests for linkage disequilibrium were checked by using the GenePop v.4.0 [62]. The total number of alleles (*N_A_*), allelic richness (*R*), gene diversity (*GD*) and allele frequencies were calculated in Fstat v.2.9.3.2 [63].

The presence of genetic bottlenecks was tested by using the heterozygosity excess method [64] implemented in Bottleneck v.1.2.02 [65]. Following recommendations of Piry et al., we used the two-phase model (TPM) with 95% stepwise mutations and a variance of 12 [65], [66]. We used the Wilcoxon signed-rank test because this test was proven to be more powerful when less than 20 loci are used [65]. The mode-shift test implemented in Bottleneck [65] was also run.

#### Population structure and migration

First, we assessed population structure by using pairwise *F*_ST_ values calculated in Arlequin v.3.11 and the Bayesian clustering approach implemented in Structure v.2.3.3 [66], [67], [68]. For analyses using Structure, the admixture model with correlated allele frequencies was chosen and the number of genetic groups K was set from 1 to 8. Each run used five replicates, which consisted of a burn-in period of 1 000 000 MCMC steps followed by 1 000 000 MCMC steps. Plots of the mean value of the log posterior probability of the data [mean of lnP(D)] were examined to check the maximum lnP(D) value [66]. Because spatially explicit Bayesian clustering methods can be powerful when inferring genetic structure [69], [70], particularly at low levels of differentiation [71], [72], we used Geneland v.4.0.3 [72], [73], [74] to search for structure in the rhesus macaque. We specified a Kmax of 8, used the correlated allele frequency model, and allowed for the presence of null alleles. This analysis included samples that had specific locations or provincial localities. The analysis was run for 1,000,000 generations and a thinning of 1,000. To make sure the MCMC was converging and mixing properly, we used 10 independent runs and compared parameter estimates. For parameter inference we selected the run with the highest log posterior probability and specified a burnin of 250.

We also used the maximum likelihood and Bayesian inference [75], [76], [77] algorithms in migrate v.3.3.2 [78] to estimate test the null hypothesis of unbiased, equal rates of migration rates and equal effective population sizes between the two largest haplogroups resolved in the haplotype network. We chose the single-step model in the analysis. For the maximum likelihood analysis, we specified three long chains while discarding the first 100,000 trees per chain. For the Bayesian inference, we employed 10 replicates while also discarding the first 100,000 trees per chain as burn-in.

## Results

### Sequence Data

The D-loop-only dataset included 168 variable (polymorphic) sites of which 26 were autapomorphic and 142 potentially parsimony informative. Inclusion of 16 indels resulted in 214 haplotypes, 92 of which we generated and 122 downloaded from GenBank. The haplotypic composition of each sampled population was summarized in Table S1. The concatenated dataset contained 2,260 nucleotide positions in 67 ingroup haplotypes plus 12 in the outgroup. About 29% of the positions (660 bp) were variable and 462 were potentially parsimony-informative. No evidence of NUMTs was detected.

### Genealogical Relationships

**Figure 2:**
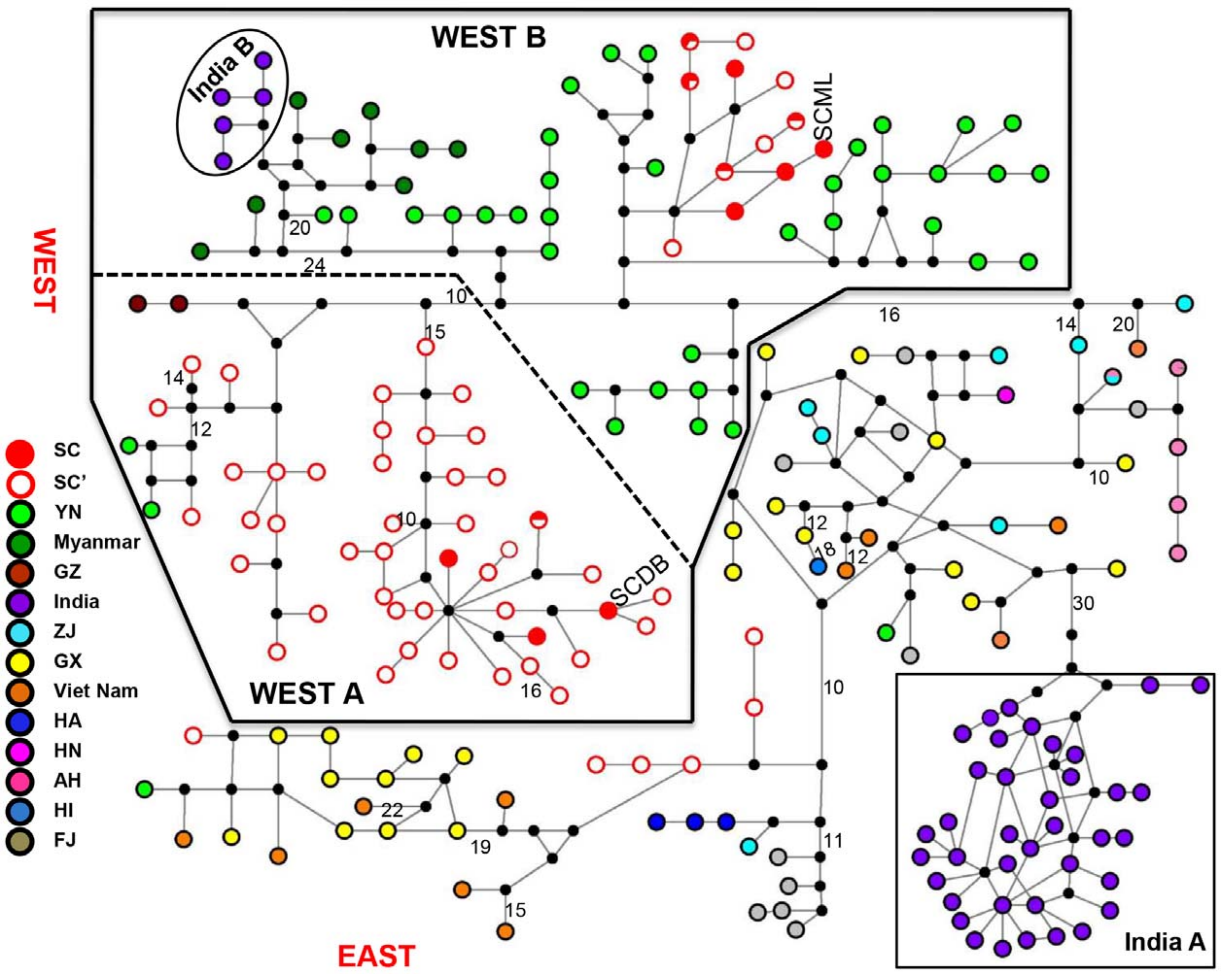
Median-joining network for 214 haplotypes from 735 rhesus macaques, *Macaca mulatta*, based on 489 bp of mtDNA D-loop. The size of each circle is not proportional to the observed frequency of a given haplotype. Closed black circles are inferred intermediate haplotypes not sampled in our study. The largest possible number of mutational steps between two haplotypes is shown.

Analysis of the D-loop data did not yield a well-resolved MP tree (Figure S1) whereas the haplotype network exhibited considerable substructure (Figure 2). The two major haplogroups of Chinese rhesus macaques identified in previous studies [9], [24]–WEST and EAST–were recovered in the network. Sixteen mutational steps separated these two clusters. The network further delineated two discrete subhaplogroups within WEST: WEST A and WEST B. WEST A, included 45 haplotypes from Sichuan, two from Yunnan, and two from Guizhou, and corresponded to subhaplogroups ChiW2 (100% support) and ChiW3 (65% support) reported by Smith and McDonough [9], and Haplogroup A (100% support) reported by Li et al. [24]. Subhaplogroup WEST B, which mainly corresponded to subhaplogroup ChiW1 and Ind2 [9] and haplogroup B [29], was comprised of 57 haplotypes, including 35 from Yunnan, 12 from Sichuan, five from India B, and eight from Myanmar.

Haplogroup EAST mainly corresponded to haplogroup ChiE and Ind1, defined by Smith and McDonough [9], and to haplogroups C, D, and E, reported by Li et al. [24]. Among all haplogroups from Viet Nam, and East, West, and South China, EAST was most widespread geographically and contained the greatest amount of genetic diversity. It was comprised of 11 haplotypes from Viet Nam, 11 from Fujian, eight from Zhejiang, 21 from Guangxi, five from Anhui, three from Henan, one from Hainan, one from Hunan (one sequence), two from Yunnan, and six from Sichuan provinces. EAST did not have distinct subgroups and most haplotypes were restricted to one locality. Populations in both Hainan and Hunan had multiple samples yet only one haplotype. The 38 haplotypes from India comprised a unique haplogroup, which was separated by 30 mutational steps from nearest haplogroup EAST. Because of lack of specific locality information for the Indian populations, we could not conduct further analyses. Regardless, evidence suggested that the more widespread Indian and Burmese rhesus macaques contained greater genetic variability than the more geographically restricted populations from western China, as reported by Smith and McDonough [9].

**Figure 3:**
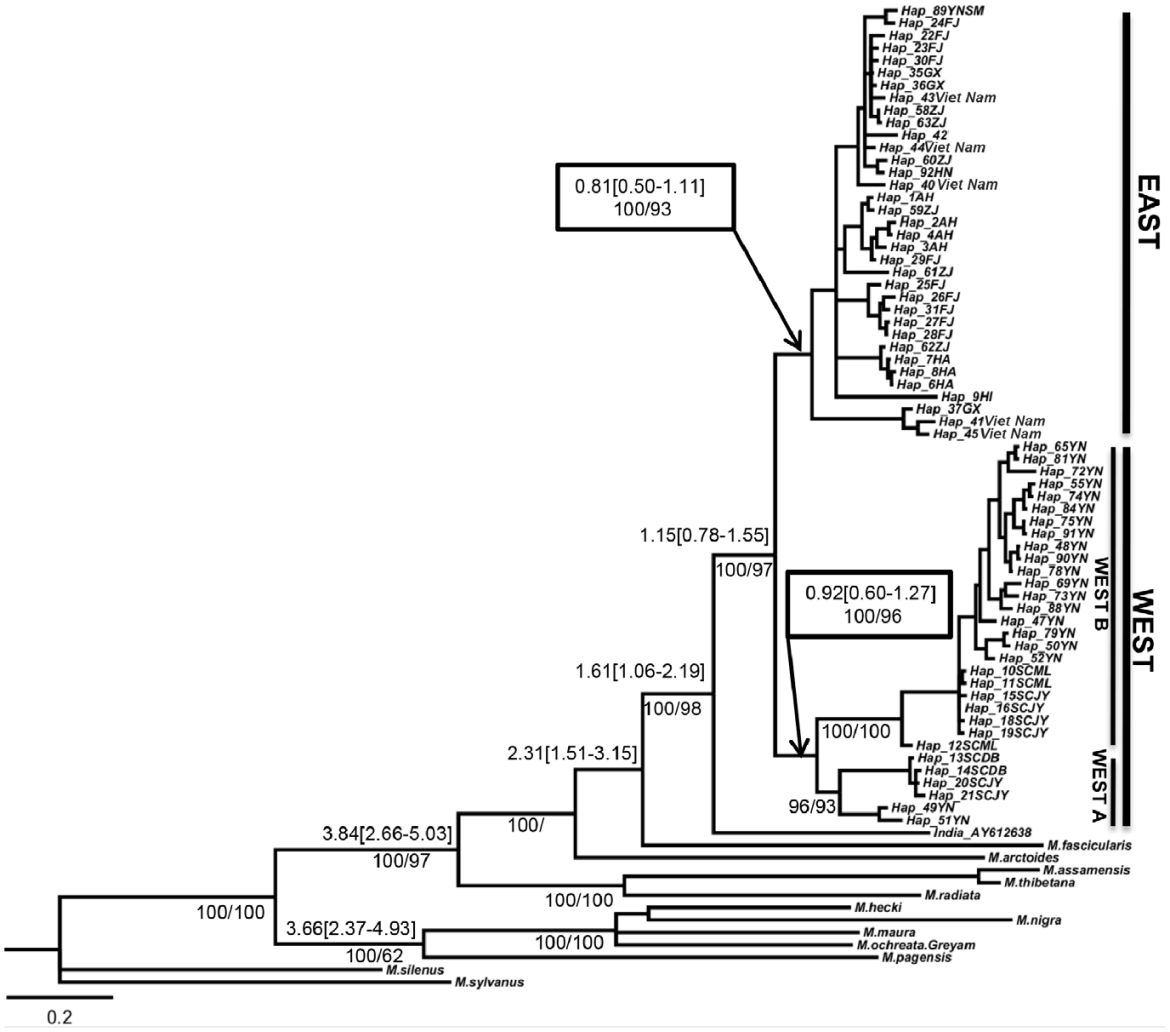
Bayesian inference matrilineal genealogy for the rhesus macaque, *Macaca mulatta*. The genealogy is based on the concatenated sequences of three mtDNA gene sequences (16S+cytb+D-loop) consisting of 2260 bp. For the main nodes, Bayesian posterior probabilities (BPP≥90 retained) and bootstrap support from maximum parsimony (MP; 1000 replicates; MP≥50 retained) are shown above the branch, respectively. And the main nodes were dated by Bayesian inference using a Yule prior and the estimates are given below the branch, with 95% confidence intervals (CI).

For the concatenated data, the Bayesian inference and maximum parsimony genealogies had identical topologies and similar support values (Figure 3). The hypothesized genealogy clearly resolved well-supported WEST and EAST haplogroups of Chinese *M. mulatta*. The WEST haplogroup was further divided into two subhaplogroups. Subhaplogroup WEST B was represented by 21 haplotypes from Yunnan and Sichuan (YNSM, YNZY, YNJG, YNMJ, and SCML) and four others were from Sichuan (SCJY). Subhaplogroup WEST A, from Sichuan and Yunnan, had two haplotypes from SCDB, two from SCJY, and two from Yunnan. Unfortunately, the two samples from Yunnan in WEST A and all the samples from SCJY were missing specific locality data. EAST was comprised 35 haplotypes, which included all sampling locations other than Yunnan and Sichuan. One haplotype from Yunnan (YN) (Hap_89YNSM) clustered in EAST.

### Isolation-by-distance and Historical Population Analysis

**Figure 4:**
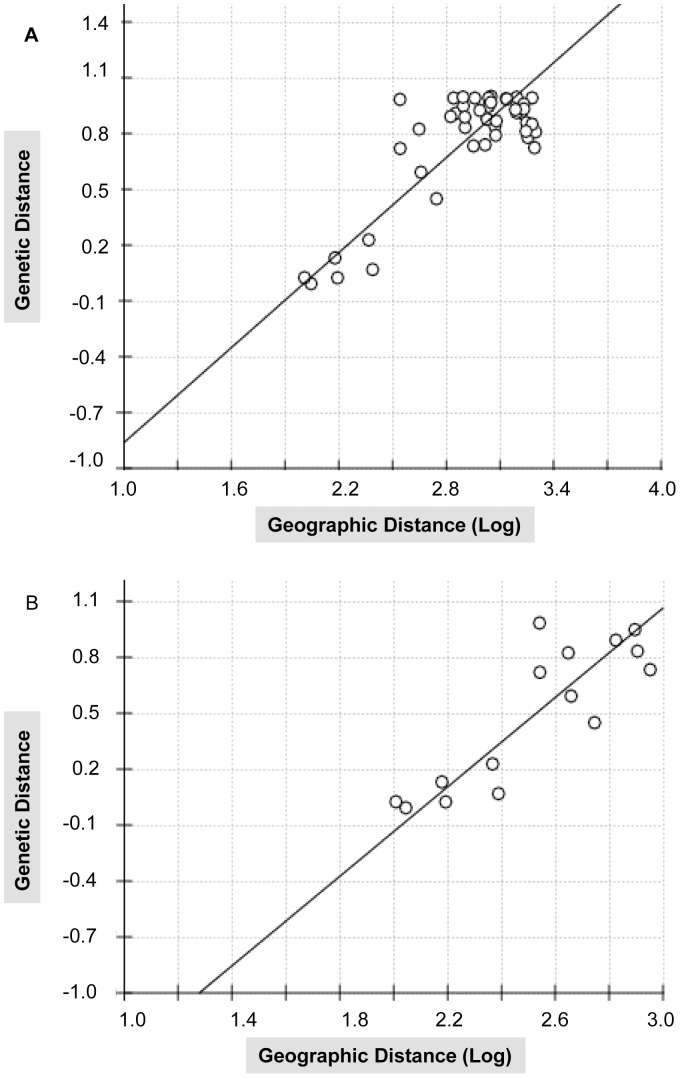
An isolation-by-distance analysis for Chinese rhesus macaques, *Macaca mulatta*. Plots of genetic distance (*F_ST_*) and log10 transformed geographical distance between the tem populations that had specific sampling information. A denotes the ten populations that displayed a significant relationship (P<0.001; R = 0.8137 with 10,000 permutations); B indicates the six western populations that displayed a significant relationship (P = 0.0188; R = 0.8527 with 10,000 permutations).

Although we had relatively few populations with specific sampling information, a positive correlation between geographical distance and genetic differentiation was obtained. The average pairwise *F_ST_* estimates based on the D-loop sequence data from the ten populations of *Macaca mulatta* that had specific sampling locality information was 0.769. The two populations from Sichuan had a far larger *F_ST_* value (0.984) compared with the four populations from Yunnan (*F_ST_ = *0.0784). IBD was observed for both all the ten populations and the six western populations that had sampling data (Figure 4A, B). The correlation between geographical distances and genetic differentiation was statistically significant for both comparisons (Mantel r = 0.8137; P<0.001 with 10,000 permutations; Mantel r = 0.8527; P = 0.0188 with 10,000 permutations, respectively). We did not calculate IBD using STRs because of the results from structure (below).

**Figure 5:**
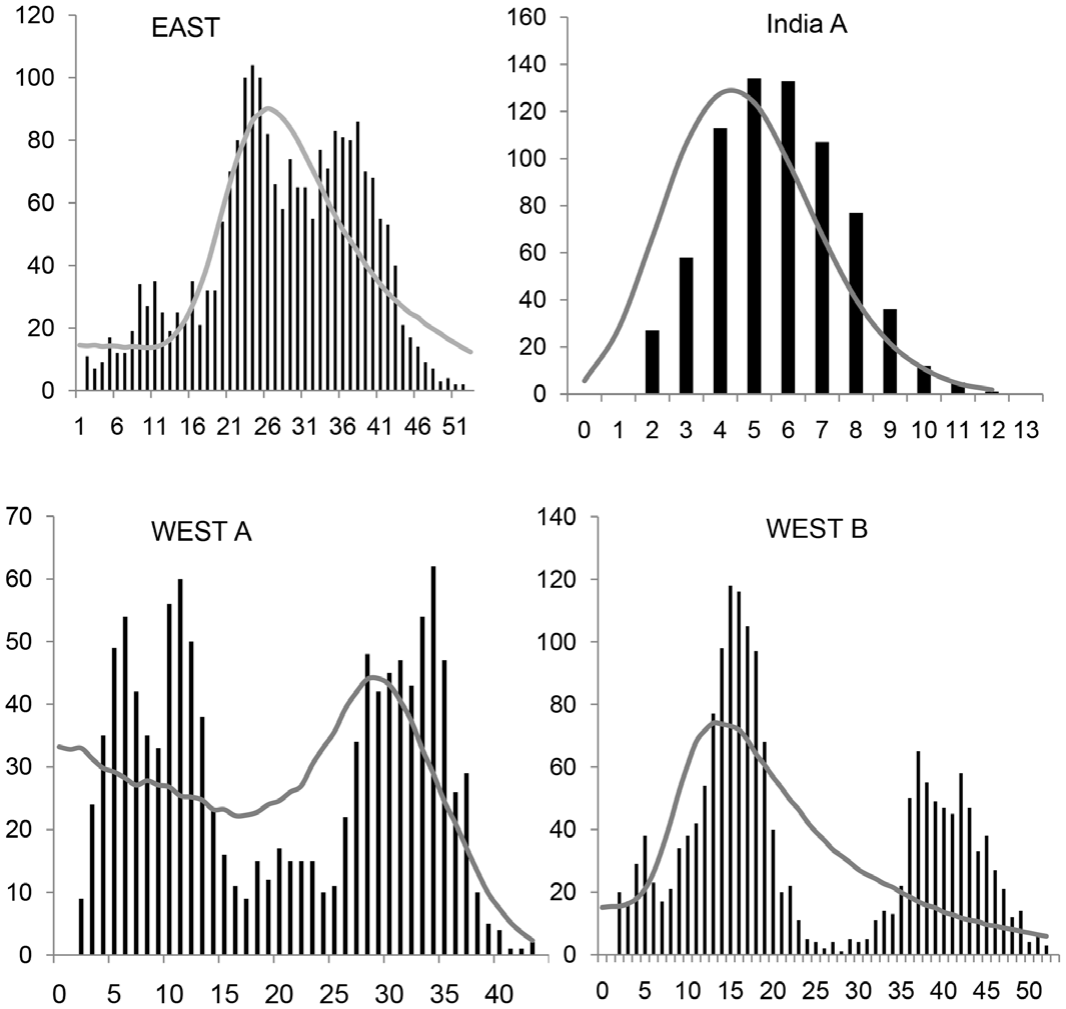
Mismatch distributions of Chinese rhesus macaques, *Macaca mulatta*, for haplogroups EAST, India A, WEST A, and WEST B depicted in **Figure 2**. The abscissa shows the number of pairwise differences between compared haplotypes. The ordinate shows the proportion for each value. Black bars show observed frequency distributions while the curves show the distribution expected under the sudden-expansion model.

Historical demographics for WEST A, WEST B, India A, and EAST provided important insights into the history of *M. mulatta* (Figure 5; Table 3). WEST A and WEST B had multimodal distributions with steep curves and a high frequency of diverged haplotypes, indicating that mismatch distributions deviated from expectations of expansion. Population expansion was indicated both within EAST and India A. Significant population growth for the four haplogroups was not inferred by neutrality tests, except for India A (Tajima’s *D* = −1.6218; *P* value = 0.0330). For the main populations of Chinese *M. mulatta*, there was considerable variation in haplotype diversity (*h*) and it ranged from 0 to 1.0000±0.063 (Table S2). Similarly, nucleotide diversity (*π*) ranged from 0 to 0.06052±0.00521. These values were highest in ZJ, FJ, GX, and Viet Nam, a reflection of the sympatric occurrences of multiple haplotypes. In addition to the haplotypes from above, four provinces occupied central locations in EAST suggesting that these haplotypes had a greater heterogeneity and an earlier history of divergence than other groups in EAST. The very low haplotype and nucleotide diversity in HA and AH corresponded to their peripheral locations in the median-joining network (Figure 2).

**Table 3:** Statistics of genetic diversity, neutrality test, and the mismatch distribution analysis.

Haplogroup/Subhaplogroup	Tajima’s *D*(*P* value)	Fu’s *Fs*(*P* value)	SSD(*P* value)	Raggedness index(*P* value)
WEST A	−0.350(0.394)	−24.175(0.000)	0.007(0.803)	0.003(0.992)
WEST B	0.290(0.695)	−24.100(0.000)	0.011(0.248)	0.002(0.855)
EAST	−0.167(0.513)	−24.076(0.001)	0.002 (0.520)	0.001(0.986)
India A	−1.622(0.033)	−25.612(0.000)	0.001(0.628)	0.018(0.457)

These analysis involved the four haplogroups of *Macaca mulatta* in China. SSD = Sum of Squares Deviations.

### Estimated Divergence Times

The main nodes (Figure 3) were dated with 95% confidence intervals. The split between *M. mulatta* and *M. fascicularis* was dated at 2.31 Ma (1.51–3.15), and the separation of the western and eastern haplogroups was dated to 1.15 Ma (0.78–1.55). The age of the most recent common ancestor (MRCA) of the western haplogroup was estimated at 0.92 Ma (0.6–1.27) while that of the eastern haplogroup was ca. 0.81 Ma (0.5–1.11).

### STR Data

**Figure 6:**
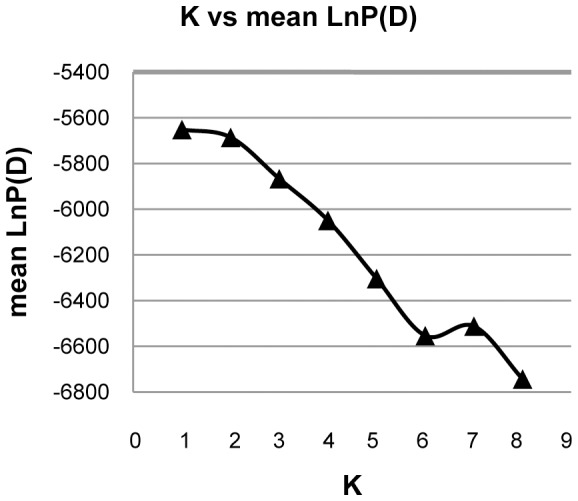
Summary diagram of Structure analyses for the rhesus macaque, *Macaca mulatta*. The analysis is based on six variable microsatellite loci for animals sampled across China.

**Figure 7:**
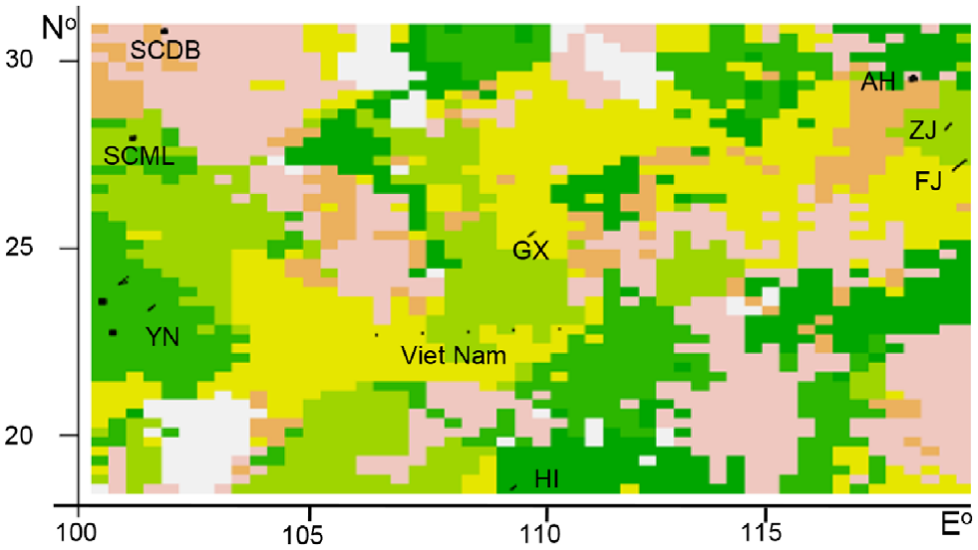
Population structure inferred in geneland at K = 7. The black dots or lines indicate the sampling locations. The abscissa and ordinate show the coordinates of sampling locations.

A total of 265 contemporary samples were genotyped at six autosomal microsatellite loci (Table S3). All microsatellite loci were located on different chromosomes and the chromosome numbers of the STRs in *M. mulatta* were found by e-PCR in the UniSTS database of NCBI. All loci were unlinked and showed no departures from Hardy-Weinberg equilibrium. The total number of alleles (*N_A_*), number of private alleles (*N_PA_*), allelic richness (*R*), and gene diversity (*GD*) were summarized in Table 4. The highest *N_A_*/locus was found in population YNJG (*N_A_*/locus = 9.5), and the highest allelic richness and gene diversity were found in population SCJY (*R* = 5.078; *GD = *0.822). In contrast, populations HI and SCDB had the lowest values [*N_A_*/locus (HI) = 3.7; *R* (HI) = 3.667; *GD* (SCDB)* = *0.677]. Using the Wilcoxon signed-rank test, we detected an excess of heterozygotes in three populations [*P*_(YNMJ)* = *_0.039; *P*_(SCJY)_ = 0.039; *P*_(HI)_ = 0.016]. Except for four populations (YNZY, FJ, HI, and SCJY), the allelic frequencies of nearly all the populations fit the normal L-shaped distribution in the mode-shift test. These results indicated that most populations had not experienced bottlenecking. Pairwise *F_ST_* values (Table 5) indicated no significant genetic differentiation between all areas sampled (mean pairwise *F_ST_* = 0.033). Structure resolved one unique genetic group for *M. mulatta* (K = 1; Figure 6). Conversely, all ten independent runs of Geneland based on all samples obtained a K-value of 7, suggesting that the MCMC was mixing well (Figure 7). Although K = 7 was the most likely number of clusters based on the run with the highest probability, K = 6 was also supported by the data and no individual was placed into cluster 7 with high probability. However, in many cases the posterior probability (PP) value for membership in a given population was not high, indicating the absence of strong support for inclusion in one population over another. All 102 samples from Yunnan (49 from YNJG, 36 from YNSM, 7 from YNMJ, and 10 from YMZY) composed one population and with the largest posterior probability (PP = 0.332) among all groups. The 29 individuals from SCDB formed one population (PP = 0.219) as did the 30 animals from AH (PP = 0.185). Another population was comprised of 30 individuals from SCML, 8 from ZJ, 2 from Viet Nam, plus 8 from FJ (PP = 0.2112). The fifth population contained seven macaques from GX, 7 from Viet Nam, and seven from FJ (PP = 0.210). Finally, the five samples from HI composed one population (PP = 0.189). Effective population sizes were nearly identical for WEST and EAST (Table 6). However, the migration rate from WEST to EAST was greater than from EAST to WEST in both maximum likelihood predictions and Bayesian inference.

**Table 4:** Statistical analyses for the microsatellite data.

Locality (N)	GD	NA/locus	R
WEST (192)	0.784	10.7	9.762
YNJG (49)	0.770	9.5	4.793
YNSM (36)	0.794	8.8	5.001
YNMJ (7)	0.766	5.2	4.567
YNZY (10)	0.731	5.8	4.629
YN (20)	0.775	8.0	4.866
SCML (30)	0.799	8.3	5.017
SCDB (29)	0.677	6.3	4.040
SCJY (10)	0.822	6.3	5.078
EAST (74)	0.75683	9.3	9.255
AH (30)	0.680	5.7	3.863
HI (5)	0.754	3.7	3.667
ZJ (8)	0.786	6.0	4.899
GX (7)	0.732	4.5	4.112
Viet Nam (9)	0.795	5.7	4.667
FJ (15)	0.792	6.7	4.803

Localities with sample sizes (*N*), gene diversity (*GD*), mean number of alleles (*N_A_*) per locus and allelic richness (*R*). Locality codes are defined in Figure 1.

**Table 5:** Pairwise *F_ST_* estimates based on STR data from populations of *Macaca mulatta*.

	YNSM	YNMJ	YNZY	YN	SCML	SCDB	SCJY	AH	HI	ZJ	GX	Viet Nam	FJ
YNJG	0.008*												
YNSM	−0.002	0.026*											
YNMJ	0.005	0.012	−0.001										
YNZY	−0.002	0.004	−0.002	−0.002									
YN	0.008*	0.023*	0.004	0.026*	0.012								
SCML	0.053*	0.070*	0.045*	0.049*	0.050*	0.079*							
SCDB	0.026*	0.025*	0.041*	0.042*	0.021	0.013	0.077*						
SCJY	0.080*	0.084*	0.123*	0.116*	0.082*	0.099*	0.117*	0.123*					
AH	0.010	0.013	0.037	0.015	0.011	0.032	0.050*	0.053	0.073*				
HI	0.025*	0.036*	0.026	0.041*	0.022	0.018	0.065*	0.035	0.015	0.017			
ZJ	0.041*	0.038*	0.018	0.045*	0.002	0.033*	0.072*	0.067*	0.129*	0.070*	0.061*		
GX	−0.008	0.001	0.008	0.014	−0.013	0.006	0.063*	0.023	0.067*	−0.005	0.010	0.034	
Viet Nam	0.009	0.020*	0.026	0.035*	0.006	0.018*	0.075*	0.038*	0.029*	0.006	0.005	0.022	−0.012

Asterisks indicate statistical significance (α = 0.05) based on sequential Bonferroni correction for multiple tests [92].

**Table 6:** Summary of the effective population sizes and migrate rates for Chinese rhesus macaques.

	Bayesian Analysis				Maximum likelihood		
Parameter	2.50%	Mode	97.50%	Mean	2.50%	MLE	97.50%
Θ_west_	0.090	0.098	0.100	0.094	1.130	1.182	1.237
Θ_east_	0.073	0.098	0.100	0.090	1.115	1.201	1.294
M_east>west_	71.333	115.000	194.000	128.052	1.617	1.981	2.396
M_west>east_	207.333	341.000	516.667	349.862	1.919	2.374	2.902

## Discussion

### Genetic Structure of Chinese *Macaca mulatta*

Using a large dataset of maternally inherited mtDNA gene sequences and nuclear DNA microsatellite data [15], [16], [17], [20], [23], [79], we discover different genetic patterns for *M. mulatta*. The large *F_ST_* value from the mtDNA data indicates a high level of genetic differentiation among populations of *M. mulatta*. Two maternal super-haplogroups exist, one in western China (Yunnan, Sichuan) and the other in eastern China (e.g., Hainan and Henan). The lineages have high levels of genetic divergence between them, as previously reported in molecular and morphological studies. For the first time and in contrast to haplogroups, our analyses of the microsatellite data do not detect significant population differences (mean pairwise *F_ST_* = 0.033; K from structure = 1; Figures 6, 7), which may suggest that the dispersal history of *M. mulatta* is too recent for the accumulation of genetic difference among the populations. This corresponds to the date of divergence between Indian and Chinese rhesus macaques estimated from SNPs at 162,000 YBP [33]. However, the conflict between the matrilineal history and the absence of nuclear DNA structure may also be due to sex-biased behavior. Male rhesus macaques are far more mobile than females and they often leave one troop to join another [37]. Because genetic recombination and sorting may obscure dispersal patterns, we mainly discuss the history of dispersal in Chinese *M. mulatta* from the perspective of females.

For the wild population of the Chinese rhesus macaque, we expect IBD because distances between the sampled populations exceed 2000 km. The significant overall IBD suggests that geographic structuring owes to geographic proximity of the samples alone, i.e. that the populations have been present and stable for a long period of time. However, EAST and WEST exhibit different structural patterns. Specific locality data are available for six western populations and an IBD analysis of these data also detects significant IBD. Thus, we cannot reject the null hypothesis of IBD.

Two relatively independent associations of matrilines occur in Yunnan and Sichuan (Figure 3): WEST B and WEST A. Only ca. 350 km separates the two populations from Sichuan (SCML and SCDB) and geographically they are closer to one another than either is to samples from Yunnan. However, these two populations exhibit the greatest amount of genetic divergence among all western localities and they do not appear to be part of the same gene pool (*F_ST_* = 0.9837). The haplotype network (Figure 2) is congruent with this discovery.

Geographical effects other than IBD are also clear. Locality SCML contains three haplotypes which includes an endemic haplotype and two haplotypes shared with other areas. All of these haplotypes are associated with a more diverse suite of subhaplogroup WEST B in Yunnan. In contrast, SCDB contains only two haplotypes, one of which consists of a single individual. Further, the haplotypes of SCDB are located in the subhaplogroup WEST A, which comprise the main haplotypes of Sichuan (mainly from Heishui, Xiaojin, and Jiulong counties) as reported by Li et al. [24]. Thus, the current distribution of *M. mulatta* in Sichuan may be a consequence of two dispersal events, which is analogous to the dispersal history of India rhesus macaques [9]. SCDB, which is one of the populations in northwestern Sichuan, represents the first dispersal. The second dispersal involves south-central and eastern Sichuan (e.g. SCML). The topography of western Sichuan involves high mountains and large rivers, and this has probably played an important role in the two dispersal events via limiting gene flow and fostering genetic drift, both of which increase the pairwise genetic distances between populations. In this case, IBD based on straight-line distances fails to detect the pattern. For WEST, some geographic structuring must occur apart from that associated with haplogroup IBD. A rapid population expansion might indicate a historical localized extirpation. However, for WEST, we do not detect significant population growth from either the mismatch distribution analysis or the neutrality test.

The matrilineal pattern in EAST differs from that of WEST. A meaningful analysis of IBD is not possible because of only four specific sampling sites. Notwithstanding, the 35 haplotypes have a different pattern and this rejects the null hypothesis of all populations being equal. Samples from Zhejiang, Fujian, and Guangxi (China) and Viet Nam contain a large mix of haplotypes (Figure 2). Nearby provinces do not possess exclusive haplogroups. Here, haplotypic diversity is extreme. For example, 21 of 26 sampled individuals in Guangxi have unique haplotypes, as do 11 of 12 individuals from Viet Nam. This pattern differs drastically from that observed in WEST, where multiple individuals have the same haplotype. In addition, haplotypes from these four provinces occupy a central location in the EAST haplogroup suggesting greater heterogeneity and an earlier history of divergence (Figure 2) than for the WEST. Hunan (one haplotype), Hainan (one haplotype), Anhui (four haplotypes), and Henan (three haplotypes) occupy peripheral locations in the haplotype network, suggesting they could be founded by recent dispersal events following the last glacial maximum (LGM). However, the former two samples are from closed, protected populations, and the latter includes three localities. Thus, our sampling strategy is likely responsible for the absence of mixed haplotypic groups on a province-wide basis, with the possible exception of the highly diverged haplogroup on Hainan Island. Highly diverged haplogroups of treefrogs also occur on Hainan Island [80].

Our structure analysis of microsatellite data does not detect significant population structure (Figure 6). This result is congruent to the spatially explicit clustering model in geneland. Although geneland analyses resolve a K of 7, the posterior probability values for different population memberships are not high enough strongly support inclusion of an individual in one population over another. Table 6 yields two points: the effective population sizes of WEST and EAST are nearly identical and thus we cannot reject the null hypothesis. This suggests that colonization of the two regions may have occurred at the same time scale, which corresponds to the time estimates (Figure 3). Further, analyses using migrate reject the null hypothesis of unbiased migration. Migration and historical gene flow occurred predominately from WEST to EAST versus EAST to WEST, which suggests dispersal is more common in lowland areas than highlands. Pleistocene climatic cycling may drive the latter case.

### Estimated Divergence Times and Dispersal

We estimate the matrilineal split between *M. mulatta* and its sister species *M. fascicularis* at 2.31 Ma (1.51–3.15, 95%). This date is similar to prior estimates of 2.6 Ma [20] and 2.5 Ma [17]. The MRCA of *M. mulatta* dates to 1.61 Ma (1.06–2.19, 95%), which is congruent with the 1.2 Ma in a previous study [17]. We estimate the age of the MRCA of WEST at 0.92 Ma (0.60–1.27, 95%) and for EAST to be 0.81 Ma (0.50–1.11, 95%). This time estimate differs substantially from the date of divergence between Indian and Chinese rhesus macaques estimated from SNPs as being 0.16 Ma [28]. The comparisons of splits within *M. mulatta* are not equivalent for three reasons: they occur for different events; one is based on a single locus and the other on multiple loci; and our estimate is for the historical split of the matrilines only and we note that our study demonstrates different sex-biased patterns. Perhaps most important, the SNP data estimate the cessation of biparental gene flow between the two groups as mediated by male dispersal. Thus, it is likely the difference reflects inheritance mechanisms of the two genomes, i.e. the maternally inherited mtDNA genome versus nuclear biparentally inherited SNPs and male-mediated gene flow.

**Figure 8:**
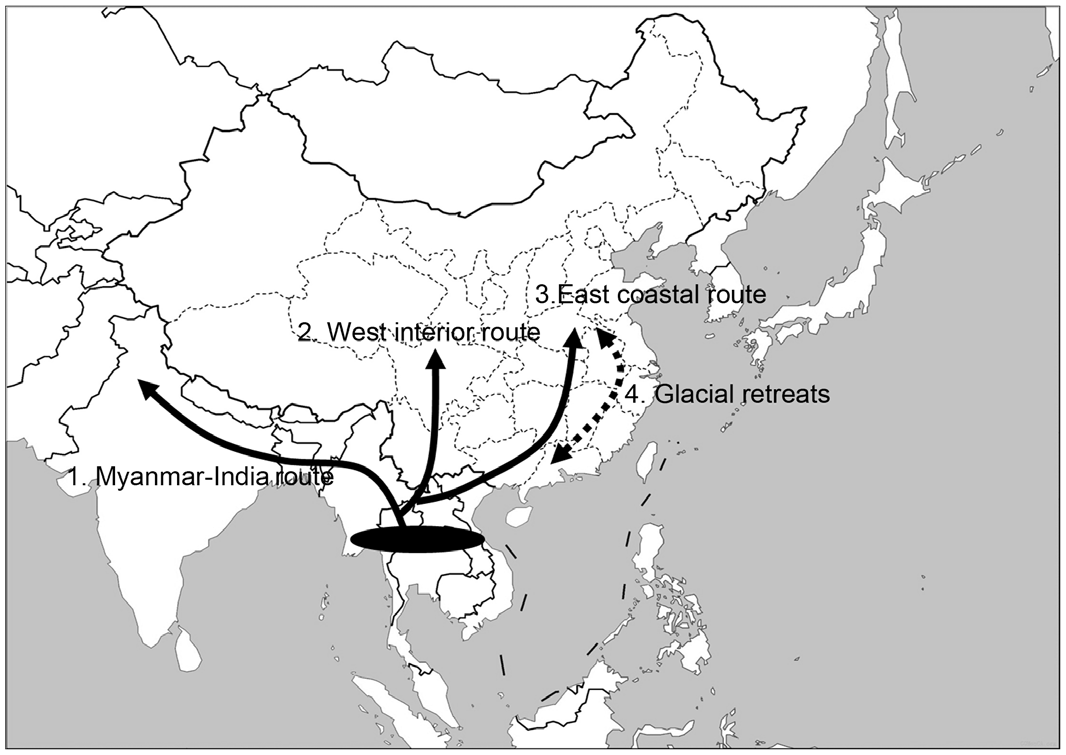
Biogeographic scenario for *Macaca mulatta*. The species originated in or near the northern part of the Indochinese peninsula, probably around 2.31 Ma. Two different dispersal routes occurred during a rapid northward expansion: the Myanmar-India route and Viet Nam-China route. Pleistocene glaciation may have affected the eastern haplogroup more than the western haplogroup.

Our time estimates yield a possible scenario of the dispersal history for *M. mulatta*. *Macaca mulatta* likely diverged from its ancestor shared with *fascicularis* about 2.31 Ma in or near northern Indochina. About 1.61 Ma (1.06–2.19, 95%), *M. mulatta* appears to have become widely distributed via a northward expansion using two different dispersal routes: Myanmar–India and Viet Nam–China (Figure 8). Dispersal into Myanmar and the Indian subcontinent is likely to have occurred along the Bay of Bengal. The second route appears to follow the eastern coast, arriving first in northern Viet Nam followed by China. Subsequent divisions are responsible for the formation of WEST and EAST.

Haplogroups WEST and EAST appear to have diverged about 1.15 Ma (0.78–1.55, 95%), a time of great change in Chinese and Southeast Asian environments. The last orogenic push of the QTP is associated with the formation of Asian monsoons, the beginning of Chinese loess (aeolian sedimentary deposits), and the appearance of the Yellow River [1]. These changes are likely to have influenced the distribution and, thus, the genetic structure of Chinese rhesus macaques.

During the Quaternary Period, when climatic deterioration compressed suitable habitats southwards, *M. mulatta* seems to have dispersed southwards and repeatedly supplanted local matrilines. This scenario best explains the great extent of mitochondrial DNA sequence differentiation between WEST and EAST. During the Pleistocene, two refugia might have existed: the western montane areas of southwestern China, and montane eastern China [81], [82]. These two refugia may have promoted differentiation between matrilines WEST and EAST.

Glaciation appears to have more severely affected EAST than WEST. The environment of East China experienced dramatic changes during the Quaternary [83]. The permafrost of North China expanded southwards by about 10° N latitude and reached the location of the Great Wall. The mean annual temperature lowered about 10°C to 12°C. The ice-age “mammoth fauna” roamed northern China southwards to the estuary of the Yangtze River. As sea-levels lowered about 140 m at times of maximum glaciation, the shoreline expanded eastwardly about 600 km [83].

We detect multiple mtDNA haplogroups in Zhejiang, Fujian, and Guangxi, China and in Viet Nam. The absence of overall geographic structuring and the apparent absence of IBD may have resulted from either the southward retreat of multiple haplogroups to a refuge during glacial periods, or a population expansion during interglacial periods [18], [29]. However, it is also possible that our sampling strategy and human translocations influenced the patterns. Explanations involving natural environmental changes and anthropogenic influences have advantages. The former may more reasonably explain population expansions, as indicated by our mismatch distribution analysis. And the latter may provide a better explanation given the long, traditional trade in rhesus macaques in coastal regions of southeastern China, especially in Guangdong.

### Implications for the Conservation of *M. mulatta* in China

The most serious threats to *M. mulatta* come from human habitat destruction and illegal poaching. These activities are responsible for the protection of the species as listed in Category II of the Chinese Wildlife Protection Act (1989) and in Appendix II of the Convention on the International Trade in Endangered Species of Fauna and Flora (CITES) [84]. For conservation, it is imperative that taxonomy reflect genetic and historical diversity. After all, the goal of conservation is to protect the evolutionary potential of a species, i.e. diversity, and bad taxonomy kills [85], [86], [87]. Consequently, future initiatives should determine whether the genetic history of *M. mulatta* corresponds to the traditional subspecific taxonomy or not, and this initiative is underway.

The western and eastern haplogroups indicate an important level of genetic structuring. Thus, WEST and EAST might be regarded as separate evolutionarily significant units (ESUs). These groups are experiencing shrinking distributions, increasing genetic isolation, and decreasing haplotype diversity. Attention should be paid to conserving populations in Henan and Hainan because both have very low genetic diversity, small residual areas of habitat, and significant morphological differences [8], [88], [89]. Further, the population is Henan occurs at the highest latitude of this species.

## Supporting Information

Figure S1 **A maximum parsimony tree for wild Chinese rhesus macaques,** ***Macaca mulatta*** **, derived from 214 D-loop haplotypes.** Four outgroup haplotypes were used to root the tree. Bootstrap support from maximum parsimony (1000 replicates) (≥50% retained) were shown at the nodes.
(TIF)

Table S1 The distribution of 214 D-loop haplotypes in each sampling location of wild Chinese rhesus macaques, *Macaca mulatta*. The first 92 haplotypes were sequenced in this study and the last 122 haplotypes were downloaded from GenBank. The 67 haplotypes colored in red were selected for concatenation with other mtDNA genes.
(XLS)

Table S2 Statistics of genetic diversity, neutrality test and the mismatch distribution analysis. These analyses were performed for main populations of *Macaca mulatta* in China. SSD = Sum of Squares Deviations.
(DOCX)

Table S3 The STR data for the wild Chinese rhesus macaque, *Macaca mulatta*.
(XLSX)
