# Supplementary figures and images for: Ecological Genetics of Chinese Rhesus Macaque in Response to Mountain Building: All Things Are Not Equal
Source: PLoS One. 2013 Feb 6;8(2):e55315. doi: 10.1371/journal.pone.0055315 (PMC3566204; doi:10.1371/journal.pone.0055315)

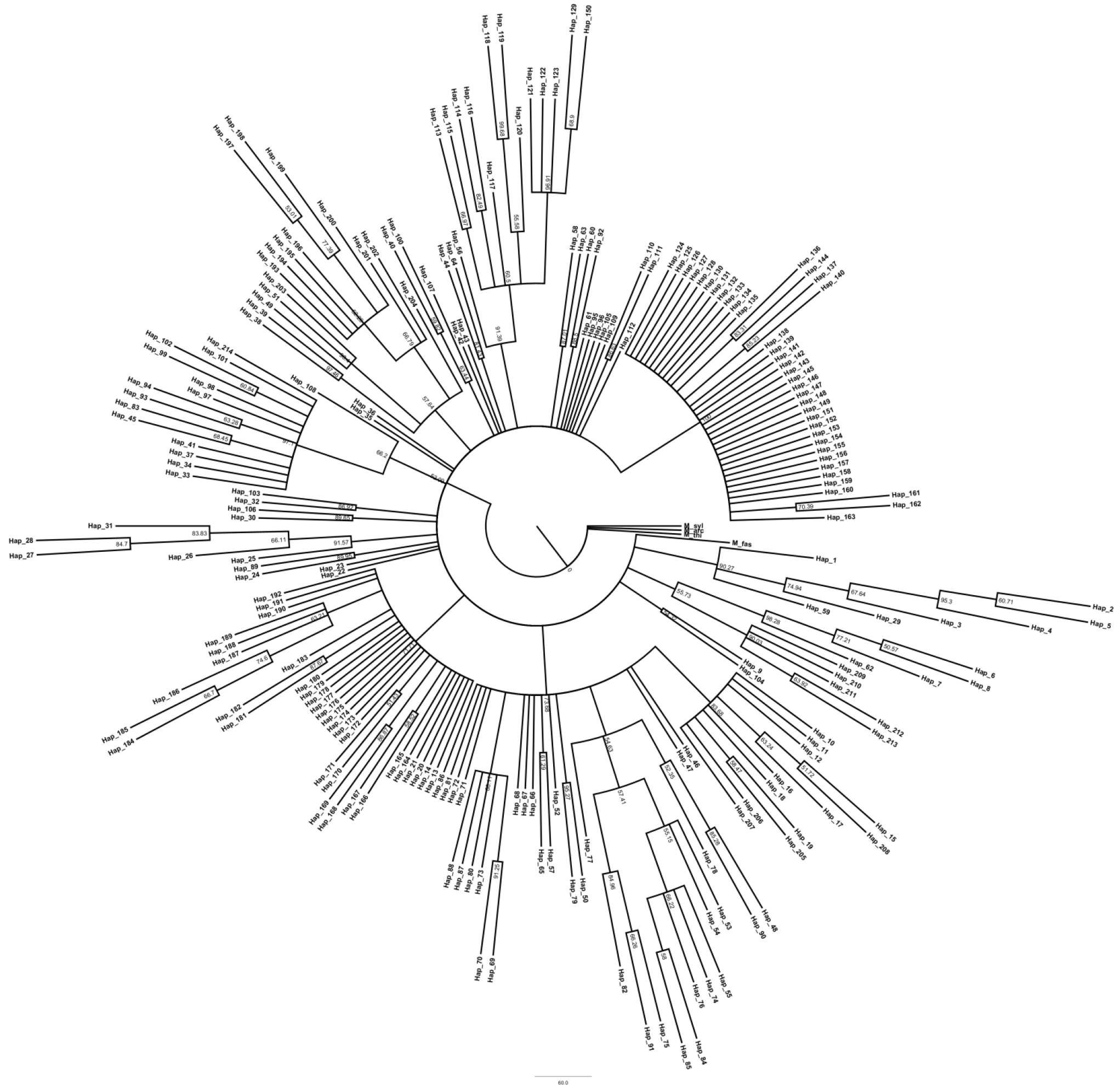

Supplement: Figure S1 — A maximum parsimony tree for wild Chinese rhesus macaques, Macaca mulatta , derived from 214 D-loop haplotypes. Four outgroup haplotypes were used to root the tree. Bootstrap support from maximum parsimony (1000 replicates) (≥50% retained) were shown at the nodes. (TIF) [file pone.0055315.s001.tif]
