# Supplementary material for: Ecological Genetics of Chinese Rhesus Macaque in Response to Mountain Building: All Things Are Not Equal
Source: PLoS One. 2013 Feb 6;8(2):e55315. doi: 10.1371/journal.pone.0055315 (PMC3566204; doi:10.1371/journal.pone.0055315)
Supplement: Table S2 — Statistics of genetic diversity, neutrality test and the mismatch distribution analysis. These analyses were performed for main populations of Macaca mulatta in China. SSD = Sum of Squares Deviations. (DOCX) [file pone.0055315.s003.docx]

**Table S2. Statistics of genetic diversity, neutrality test and the mismatch distribution analysis.** These analysis are for main populations of *Macaca mulatta* in China. SSD = Sum of Squares Deviations.

| **Lineage/ Sublineage** | **Haplotype diversity (*h*)** | **Nucleotide diversity (*π*)** | **Tajima's *D***  **(*P* value)** | ***Fs***  **(*P* value)** | **SSD**  **(*P* value)** | **Raggedness index**  **(*P* value)** |
| --- | --- | --- | --- | --- | --- | --- |
| HA(35) | 0.213±0.088 | 0.00075±0.00032 | -1.07973(0.15800) | -0.37389 (0.28800) | 0.02703 (0.15800) | 0.57319 (0.59500) |
| AH(30) | 0.637±0.068 | 0.01012±0.00090 | 1.16763 (0.90000) | 5.61655 (0.97600) | 0.17446 (0.05700) | 0.22584 (0.02600) |
| ZJ(8) | 1.000±0.063 | 0.03806±0.00537 | -0.03931(0.51000) | -0.94922(0.19900) | 0.02800(0.73000) | 0.05357(0.72800) |
| FJ(12) | 0.985±0.040 | 0.03419±0.00289 | 0.17740(0.61900) | -1.29398(0.19600) | 0.04763(0.01300) | 0.09527(0.07500) |
| GX(26) | 0.978±0.018 | 0.04205±0.00247 | 0.53890 (0.79500) | -2.51964(0.14200) | 0.00644(0.86900) | 0.00845(0.78500) |
| V(12) | 0.985±0.040 | 0.06052±0.00521 | -0.28024(0.43300) | -0.13714(0.39800) | 0.01636(0.57900) | 0.04109(0.33000) |
| YN(119) | 0.945±0.009 | 0.02428±0.00231 | -1.14307 (0.11200) | -3.36172 (0.26300) | 0.00425 (0.68500) | 0.00844 (0.45900) |
| YNSM(45) | 0.937±0.018 | 0.02801±0.00433 | --1.15563 (0.11400) | -0.35679 (0.49900) | 0.00522(0.65200) | 0.00720 (0.76400) |
| YNJG(50) | 0.882±0.028 | 0.01675±0.00178 | --0.26571 (0.44000) | 0.81288 (0.67900) | 0.02512 (0.51400) | 0.04064 (0.21000) |
| YNMJ(7) | 0.857±0.137 | 0.01420±0.00240 | 0.16377(0.56300) | 1.19017(0.65700) | 0.06456(0.23400) | 0.14286(0.42700) |
| YNZY(10) | 0.911±0.077 | 0.02244±0.01040 | -1.35750(0.08700) | 1.39203(0.73200) | 0.05396(0.29600) | 0.10469(0.28800) |
| SCML(30) | 0.641±0.044 | 0.00159±0.00018 | 1.20693(0.89000) | 1.07231 (0.70300) | 0.01329 (0.12100) | 0.14285 (0.10900) |
| SCDB(29) | 0.069±0.063 | 0.00069±0.00063 | -2.00682(0.00500) | 0.94294(0.54200) | 0.00705(0.05000) | 0.87634(0.86800) |
| SCJY(10) | 0.911±0.077 | 0.02860±0.01029 | -0.16076 (0.48100) | 1.92094 (0.80600) | 0.06251(0.28100) | 0.05827(0.70100) |
